# Effects of Rapid Palatal Expansion on the Upper Airway Space in Children with Obstructive Sleep Apnea (OSA): A Case-Control Study

**DOI:** 10.3390/children10020244

**Published:** 2023-01-30

**Authors:** Angela Galeotti, Roberto Gatto, Silvia Caruso, Simone Piga, Wanda Maldonato, Emanuela Sitzia, Valeria Viarani, Gaia Bompiani, Francesco Aristei, Giuseppe Marzo, Paola Festa

**Affiliations:** 1 Dentistry Unit, Bambino Gesù Children’s Hospital, IRCCS, 00165 Rome, Italy; 2 Department of Life, Health and Environmental Sciences, University of L’Aquila, 67100 L’Aquila, Italy; 3 Unit of Clinical Epidemiology, Medical Direction, Bambino Gesù Children’s Research Hospital, IRCCS, 00165 Rome, Italy; 4 Otorhinolaryngology Unit, Department of Specialist Surgeries, Bambino Gesù Children’s Hospital, IRCCS, 00165 Rome, Italy

**Keywords:** cephalometry, rapid maxillary expander, obstruction sleep apnea, upper airways, children

## Abstract

Obstructive Sleep Apnea (OSA) in children needs a multidisciplinary approach. Even if the first-line treatment of pediatric OSA is adenotonsillectomy, nowadays rapid palatal expansion (RPE) is considered a valid additional treatment. The aim of this study is to evaluate cephalometric changes in upper airways dimensions after rapid palatal expansion (RPE) in children suffering from Obstructive Sleep Apnea (OSA). A total of 37 children (range age 4–10 years) with diagnosis of OSA referred to Dentistry Unit of Bambino Gesù Children’s Research Hospital IRCCS (Rome, Italy) were included in this pre-post study and underwent lateral radiographs at the start (T0) and at the end (T1) of a RPE treatment. Inclusion criteria were: diagnosis of OSA confirmed by cardiorespiratory polygraphy (AHI > 1) or pulse oximetry (McGill = >2), skeletal maxillary contraction evaluated by presence of posterior crossbite. A control group of 39 untreated patients (range age 4–11 years), in good general health, was set up. A paired T-test was used to investigate the statistical differences between T0 and T1 values in both groups. The results showed a statistically significant increase of nasopharyngeal width in the treated group after RPE treatment. Moreover, the angle that identifies mandibular divergence compared to palatal plane (PP-MP°) was significantly reduced. In the control group, no statistically significant differences were observed. The present study showed that RPE treatment determines a significant sagittal space increase in the upper airways space and a counterclockwise mandibular growth in children with OSA compared to a control group. These results suggest that a widening of the nasal cavities induced by RPE may support a return to physiological nasal breathing and promote a counterclockwise mandibular growth in children. This evidence confirms the crucial role of the orthodontist in the management of OSA in pediatric patients.

## 1. Introduction

Obstructive Sleep Apnea (OSA) in children is a common breathing disorder characterized by recurrent episodes of partial or complete upper airway obstruction that disrupt the normal sleep pattern [1].

Other sleep breathing disorders include primary snoring and increased upper airway resistance syndrome (UARS) [2].

Paglia et al., in 2019 [3], reported that primary snoring affects about 27% of children, whereas 1–3% are suffering from OSA, with a peak age between 2 and 5 years, which is the age of greatest increase of adenotonsillar lymphoid tissue [4].

Brunetti et al. [5] estimated that in Italy there is a prevalence of pediatric OSA of 1.8%, especially in pre-school and school years, compared with 4.9% of habitual snoring.

The main risk factors for OSA involve adenotonsillar hypertrophy, neuromuscular disorders, obesity, and craniofacial anomalies such as deficiency in the transversal dimension of the upper jaw, macroglossia, retrognathia, hyperdivergent mandibular pattern, midface hypoplasia and anterior open bite [6,7]. Moreover, the presence of craniofacial anomalies also has a genetic component [8] that may influence the anatomical development of the upper airways [9].

Commonly, children with systemic diseases and specific craniofacial features suffer from OSA [10]. Hill et al. [11], in particular, reported that children with Down syndrome have a high incidence of OSAS, moderate to severe grade, due to the common adenotonsillar hypertrophy and reduced airway dimensions, in relation to common midface hypoplasia, small jaw and hypotonia.

A significant association was evidenced between malocclusion and OSA, in particular the prevalence of posterior crossbite, deviations in overjet and overbite was higher in OSA children [12]. 

Associated symptoms are snoring, daytime sleepiness or hyperactivity, headache, nocturnal enuresis, and restless sleep [13].

An early diagnosis is very important to prevent serious complications, such as neurocognitive impairment, behavioral, metabolic, and cardiovascular problems [1].

The diagnostic pathway requires above all anamnestic investigation to collect signs and symptoms with the use of parent-specific questionnaires [14,15]. 

Clinical examination includes pediatric, otorhinolaryngological and myofunctional evaluation [13].

Sleep examination is indispensable for the diagnosis of OSAS. The Gold Standard is nocturnal polysomnography which allows a complete examination of the patient’s sleep (PSG) [16]. 

Unfortunately, it is not always feasible to perform this examination in pediatric patients as it must be conducted in dedicated sleep laboratories. Thus, alternative methods to study the presence of episodes of apnea and/or oxygen desaturation during sleep have been considered such as cardiorespiratory polygraphy or pulse oximetry [17]. Adenotonsillectomy represents the first-line treatment in the presence of adenotonsillar hypertrophy but this procedure is not always effective [18,19]. After adenotonsillectomy, about 25% of patients retain residual obstructive apnea events during sleep [18,19]. Risk factors for residual OSA after adenotonsillectomy include: more than seven years of age, obesity, severity of sleep disorder and presence of asthma [20]. For this reason, several authors have focused attention on the therapeutic role of an orthodontic approach to obstructive sleep apnea in children [21].

The presence of palatal contraction has been demonstrated to be associated with the presence of OSA in childhood [6] so rapid palatal expansion (RPE) is commonly used in young patients to expand the upper maxilla and it is also recognized as a valid additional treatment in children with OSA [22,23].

Several studies have demonstrated the effectiveness of maxillary expansion to induce widening of the anterior part of the oropharyngeal and nasal space, and the reduction of nasal resistance to the passage of air flow [4,24].

Previous studies evidenced an increase in airway space after the use of orthodontic appliances in OSA children [4,25,26] but few studies have analyzed the upper airway changes after early palatal expansion in a large sample of OSA children.

The aim of this study, therefore, is to evaluate the cephalometric variations of the upper airways dimension after rapid maxillary expansion in children suffering from OSA.

## 2. Materials and Methods

The study was approved by the Ethics Committee of Bambino Gesù Children’s Research Hospital, IRCCS (Rome, Italy) (protocol number: 1187_2016), 15 September 2016.

### 2.1. Subjects 

A group of 139 children from a previous study [12] with diagnosis of OSA at the Otorhinolaryngology Unit of Bambino Gesù Children’s Research Hospital, IRCCS (Rome, Italy) was considered for this study. Exclusion criteria included: diagnosis of genetic syndromes, missing teeth, previous orthodontic treatment, previous adenoidectomy or adenotonsillectomy and any surgery of the upper airways. The patients underwent dental examination at the Dentistry Unit of the Bambino Gesù Children’s Research Hospital, IRCCS (Rome, Italy) by specialist in Orthodontics. Inclusion criteria were: diagnosis of OSA confirmed by cardiorespiratory polygraphy (AHI > 1) as described in a previous study [7] or pulse oximetry (McGill = >2), with at home overnight sleep studies [25,27]; skeletal maxillary contraction evaluated by the presence of posterior crossbite. 

Polygraphy was conducted with the “Somtè/Siesta” portable system (Compumedics, Australia). Parameters such as nasal pressure, tracheal sound, heart rate and pulse oximetry were recorded [7]. Pulse oximetry was conducted as reported by Brouillette et al. [25]. Pulse oximeters set for a mean time of 2 s were used for oxygen saturation (SpO2) (RAD 5, Masimo, Irvine, CA, USA). Data were processed with Profox Oximetry Software, Version Masimo 0706.05D. 

After the inclusion and exclusion criteria application, 37 children (range age 4–10 years) were selected for a rapid palatal expansion (RPE) treatment and were enrolled for this pre-post study. The parents accepted to participate the study and signed informed consent.

A control group of 39 subjects (range age 4–11 years), were collected from patients examined in the Dentistry Unit. The children were in good general health, had no systemic disease and no missing teeth. Two lateral cephalograms were performed pre and post an observation period. No orthodontic treatment and adenoidectomy or adenotonsillectomy were performed during this period.

### 2.2. Methods

#### 2.2.1. Rapid Palatal Expansion

**Figure 1:**
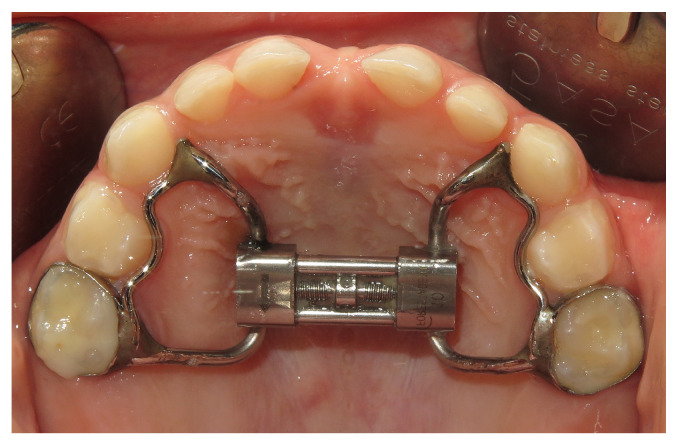
Customized rapid palatal expander (RPE).

The patients were treated with rapid palatal expander (RPE) cemented with glass ionomer cement (Fuji Ortho, GC, Cary, NC, USA). Two bands type anchored at the second deciduous maxillary molars or permanent upper first molars (Figure 1), or bite block type (Mc Namara expander) rapid palatal expander were used. The RPE is built with a central screw that is required to be activated to open the midpalatal suture. The expansion screw is placed as close as possible to the palatal vault to deliver force to the center of resistance of the maxillary molars and produce orthopedic effects on the upper jaw. The expansion was performed one turn a day (0.25 mm/turn) until the palatal cusps of the upper molars were contacting the buccal cusps of the lower molars. At the end of the expansion period the RPE was manteined in place as a retainer for 9 months. Mean duration and standard deviation of the therapy was 13.2 ± 3.6 months. 

#### 2.2.2. Cephalometric Analysis

74 lateral cephalometric radiographs, 37 at the start (T0) and 37 at the end (T1) of RPE treatment, were taken with the same cephalix cephalostat (OP 3D Pro; Kavo, Milan, Italy) at the Diagnostic Imaging Unit of the Bambino Gesù Children’s Hospital IRCCS (Rome, Italy) for the treated group. Radiographs were taken with the subjects standing, the head fixed in the cephalostat with ear and forehead supports, the teeth in their maximum intercuspal position and the head in the natural position.

39 subjects included in the control group took two lateral cephalometric radiographs at T0 and T1 with a time interval of 9.7 months to 18.6 months (mean 14.2 ± 2.8 months) by means of the same cephalix cephalostat (OP 3D Pro; Kavo, Italy) at the Diagnostic Imaging Unit of the Bambino Gesù Children’s Hospital IRCCS (Rome, Italy). Radiographs were taken with the subjects standing, the head fixed in the cephalostat with ear and forehead supports, the teeth in their maximum intercuspal position and the head in the natural position.

All lateral cephalograms were analyzed and the measurements of the distances and angles were recorded by the same operator, using computerized software “Orisceph Rx CE, 1370” (Elite Computer, Milan, Italy).

**Figure 2:**
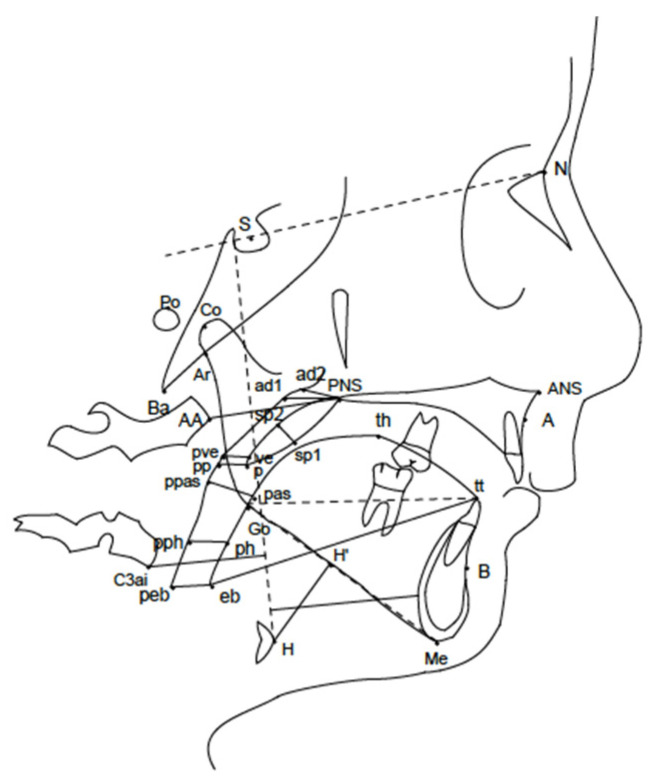
Cephalometric landmarks used for the analysis [7].

The Kirjavainen analysis [28] was used as cephalometric method to evaluate changes of vertical, sagittal and pharyngeal parameters between T0 and T1 for both the study and control groups (Figure 2). 

The technical errors of measurement were calculated from 14 randomly selected participants. One examiner revalued all measurements after a period of at least 8 weeks. The method error for the 27 measurements was calculated using Dahlberg’s formula [29]. Systematic differences between duplicated measurements were tested using a paired Student’s *t*-test with the type I error set at <0.1 [30].

### 2.3. Sample Size Calculation

The sample size was calculated considering Ad1-PNS distance: the distance of Ad1 point to posterior nasal spine (PNS). Ad1 is the intersection point of the posterior pharyngeal wall and the line from PNS to Basion (the most inferior-posterior point on the anterior margin of foramen magnum). A sample size of 37 patients treated and 39 subjects not treated in the control group achieves 90% of power to detect a difference of 3 mm between the means of the two groups with estimated group standard deviations of 6 mm and with a significance level (alpha) of 0.05 (two-sided).

### 2.4. Statistical Methods

The Shapiro–Wilk test was used to evaluate the normality of the data. Continuous variables were summarized by mean and standard deviation (SD) and 95% confidence interval of the mean (95% CI). 

A paired t-test was used to determine statistical differences between the scores before RPE treatment (T0) and after RPE treatment (T1) and between the scores at T0 and T1 in the control group without treatment.

Further analysis was performed between the cases group and control group considering gender and age. The Pearson chi-square test was used for gender comparison. Two sample t-test with equal variances was used for age comparison. The statistical significance was set at *p* < 0.05.

Statistical analyses were performed using STATA, Statistical Software Release 13 (StataCorp LP, College Station, TX, USA).

## 3. Results

37 children (20 males (54.05%) and 17 females (45.95%); mean age ± SD = 6.26 ± 1.26 years) were included in the study and treated with RPE. The control group included 20 males e 19 females (mean age ± SD = 7.36 ± 0.90 years). Cephalometric measurements and their definitions are summarized in Table 1.

All the cephalometric measurements of the treated group, at T0 and T1, and their differences are presented in Table 2.

A significant variation of the nasopharyngeal distance was found after RPE treatment: ad1-PNS (mm) increased by 3.48 mm (*p* < 0.001) and ad2-PNS (mm) increased by 1.67 (*p* = 0.003) (Table 2).

A statistically significant reduction of the PP-MP angle of 1.14° (*p* = 0.043) was evidenced after RPE treatment (Table 2).

No statistically significant changes were shown in maxillary and mandible sagittal positions (SNA angle, SNB angle, ANB angle) or for the other variables (Table 2).

The cephalometric measurements of the control group, at T0 and T1, and their differences are summarized in Table 3.

The analysis performed between case group and control group showed that the two groups are not different by gender (*p* = 0.809); a statistically significant difference in age of one year (*p* < 0.001) was found.

## 4. Discussion

The aim of this study is to evaluate the cephalometric variations of the upper airways dimensions after rapid maxillary expansion in children suffering from OSA.

Rapid palatal expansion (RPE) is considered an effective method to enlarge the upper maxilla and, due to the anatomical contiguity, to wide the floor of the nasal cavity [31], but a very debated and controversial issue is the effectiveness of RPE increasing the width of the upper airways and improving the nasal airflow [32,33].

A 2021 review with 18 articles selected [30] showed an enlargement of upper dental arch, maxillary bone and improvement of nasal breathing in the short term, after RPE treatment.

Several studies have focused on the effects of RPE on the size of airways space, using two-dimensional (2D) and three-dimensional (3D) methods [4,34,35,36,37].

A recent systematic review with meta-analysis [34] showed that the results of the studies are still very conflicting: some authors evidenced a significant association between RPE and increase in the width of the upper airways [34,35,38,39], while other authors showed no significant findings [40]. All of the studies mentioned in this review [34] were performed on children with reduced transverse diameters of the upper jaw who needed palate expansion therapy, but none of these patients were diagnosed with OSA or any other type of sleep breathing disorder.

The present study investigates the sagittal variations of upper airways dimensions after rapid maxillary expansion in children suffering from OSA. We suppose that children with OSA, who have narrower upper airways than normal subjects [41], might respond differently to palatal expansion treatment.

In the present study a significant increase of cephalometric measurements of the nasopharyngeal distance (ad1-PNS = *p* < 0.001; ad2-PNS = *p* < 0.003) was found after RPE treatment in OSA children; the increase was 3.48 mm for ad1-PNS and 1.67 for ad2-PNS. These results are consistent with recent evidence reported in a three dimensional- study [42] where the authors performed Cone Beam computed tomography (CBCT) scans of 19 OSA children (mean age of 10.5 years) before (T0) and after (T1) RPE treatment. Pirelli et al. [42] showed that the volume of total upper airways increased significantly after maxillary expansion; in detail, average increases of 9.4 mm^3^ for total upper airways volume, 7.2 mm^3^ of nasal volume and of 2.0 mm^3^ nasopharinx volume were reported. In contrast with our results, these authors reported also an increase of three dimensional oropharyngeal space [42].

As a novelty compared to this previous study, our investigation presents a larger sample of children with OSA and the analysis of sagittal airway values also in a control group. The study of the cephalometric values of the control group did not show a significant difference of the airway space comparing T0 to T1. Therefore, healthy and untreated children did not have a significant change in airway space after the observation period, while the statistical significance of the changes in the group of patients with OSA is high (*p* < 0.001). These results suggest a clear role of RPE treatment in increasing the sagittal upper airways dimensions in OSA children. Furthermore, considering that the evaluation of this study has the limit of being two-dimensional, it is conceivable that the effects are even more interesting in volumetric terms. Further three-dimensional evaluations are needed to better analyze these aspects.

The comparison between the cases group and control group showed no difference in gender. There was a statistically significant difference in age of about one year when comparing the control group (mean age ± SD = 7.36 ± 0.90 years) with the case group (mean age ± SD = 6.26 ± 1.26 years) and it can be considered in the limits of our study. 

It was not possible to perform radiographs in non-treated children for ethical reasons. We therefore used as a control group children who had undergone lateral cephalograms available in our database and who met the inclusion criteria. For this reason, there is also a slight difference in the time interval T1–T0 between cases and controls. 

However, we suggest that this statistically significant difference in age of one year between cases and controls does not influence the results of the study: in fact, in clinical terms, the respiratory symptoms related to growth become evident after nine years of age when there is a physiological reduction of the adenoid and tonsillar tissue [43,44].

Another statistically significant value in our analysis is the modification of the PP-MP angle after treatment. This reduction of 1.14 degrees may not have a great impact from a clinical point of view, but it indicates an interesting modification of growth pattern in OSA children who presented always a typical hyperdivergent mandibular growth. Several authors [6] have correlated hyperdivergent growth pattern with a reduction in nasopharyngeal space in children with OSA. It was suggested that the mouth breathing and the lower position of the tongue promote posterior mandible rotation and increased vertical growth [45].

Moreover, the reduction of the angle between the palatal plane and the mandibular plane is an interesting piece of evidence in the orthodontic field. An increase in vertical mandibular growth pattern was previously reported as a consequence of orthodontic treatment [46]; the effect of RPE on craniofacial vertical growth has long been debated and there are still conflicting opinions [46,47]. 

Therefore, the results of our study suggest that rapid palatal expansion induces skeletal and functional changes in OSA children: a widening of the nasal cavities induced by RPE increase the upper airway space in the adenoidal region; this supports a return to physiological nasal breathing and promotes a counterclockwise mandibular growth in OSA children. On the other hand, the modification of the shape of the palate also allows a more correct repositioning of the tongue which contributes to physiological mandibular growth.

The limitations of our study are:

1. The use of a two-dimensional examination, but it was not possible to use a three-dimensional analysis which exposed patients to a massive radiation dose before and after treatment. For this reason, we evaluated the changes in sagittal and vertical direction, but the modifications were not studied in a transverse direction. Nevertheless, we assume that the increase of upper airways in millimeters might be much more substantial with 3D evaluation which analyzes the entire volume, as suggested by other studies [40,41].
2. No home sleep study was performed after RPE treatment; therefore, this study does not provide data on the efficacy of orthodontic therapy.
3. This study did not present long-term data.

The multidisciplinary diagnostic path is essential in pediatric OSA to attribute the right role to each of the risk factors involved in the etiology of pathology and define the better therapeutic approach.

Orthodontic treatment alone may not be sufficient to solve the sleep breathing disorder. Some studies have shown that the apnea index (AHI) decreases after RPE [48,49], while other studies have reported results that are still unclear [22]. An interesting study evidenced that the combined treatment of adenotonsillectomy and RPE were required to solve OSA in a pediatric sample [50].

Thus, the contribution of different specialists involved in the management of sleep disorder in children is important. The orthodontist plays a crucial role because the craniofacial skeletal changes and functional modification are key aspects in solving sleep respiratory breathing problems in children.

## 5. Conclusions

In conclusion, our study showed that RPE treatment has significant effects on nasopharyngeal dimensions and mandibular growth in pediatric patients with OSA compared to a control group of untreated children.

Therefore, our results suggest that a widening of the nasal cavities induced by RPE may support a return to physiological nasal breathing and promote a counterclockwise mandibular growth in children. This evidence confirms the crucial role of the orthodontist in the management of OSA in pediatric patients.

## Figures and Tables

**Table 1:** Definitions of cephalometric measurements [7].

Nasopharynx	
ad1-PNS	The distance of ad1 point to PNS (posterior nasal spine). Ad1 is the intersection point of posterior pharyngeal wall and the line from PNS to Basion (Ba)
ad2-PNS	The distance of ad2 point to PNS. Ad2 is the intersection point of posterior pharyngeal wall and the line from the midpoint of the line from sella (S) to basion (Ba) to PNS
Oropharynx	
p-pp	The distance of the tip of soft palate (p) to horizontal counterpoint on posterior pharyngeal wall (pp)
pa	The distance of the intersection points on anterior and posterior pharyngeal wall of the line from supramentale (B) to gonion (Go)
Hyoid bone	
H-H′	The distance from the most anterior and superior point of hyoid bone (H) perpendicular to mandibular plane (MP).
Maxilla	
SNA	The angle sella (S) to nasion (N) to subspinale (A)
Mandible	
SNB	The angle sella (S) to nasion (N) to supramentale (B)
ANB	The angle subspinale (A) to nasion (N) to supramentale (B)
Facial heights	
S-Go	The distance from sella (S) to gonion (Go)
N-Me	The distance from nasion (N) to menton (Me)
P-A face height (S-Go/N-Me)%	The ratio between S-Go/N-Me
Typology	
SN-MP	The angle nasion (N) to sella (S) to mandibular plane (MP).
PP-MP	The angle PP to MP. PP is the line from anterior nasal spine (ANS) to PNS
Cranial base	
SN	The distance from sella (S) to nasion (N)

**Table 2:** Cephalometric variables in treated group at time T0 and T1, expressed as mean and standard deviation (SD) and their 95% confidence interval (95% CI).

Cephalometric Variables	Time						Mean Difference by Time	*p*-Value
	T0			T1			T1–T0	
	Mean	SD	95% CI	Mean	SD	95% CI		
SNA (°)	80.9	4.43	79.4–82.4	80.81	4.76	79.2–82.4	−0.07	0.866
SNB (°)	75.6	3.97	74.3–76.9	75.8	3.9	74.5–77.2	0.26	0.492
ANB (°)	5.29	3.1	4.2–6.3	4.96	2.3	4.2–5.7	−0.32	0.381
SN (mm)	58.3	5.8	56.4–60.3	59.96	4.3	58.5–61.4	1.63	0.136
SN/MP (°)	38.3	5.8	36.4–40.3	37.9	6.0	35.9–39.9	−0.46	0.320
PP/MP (°)	32.1	5.6	30.2–33.9	30.9	5.3	29.2–32.7	−1.14	0.043 *
P-A face height (S-Go/N-Me) (%)	61.5	5.2	59.8–63.2	61.9	5.3	60.2–63.7	0.47	0.299
ad1-PNS (mm)	AD	5.6	9.2–12.9	14.6	5.6	12.7–16.5	3.48	<0.001 *
ad2-PNS (mm)	7.5	3.6	6.3–8.6	9.1	3.9	7.8–10.5	1.67	0.003 *
p-pp (mm)	13.3	4.2	11.9–14.7	13.0	4.3	11.6–14.5	−0.25	0.795
pas-ppas (mm)	14.3	3.6	13.1–15.5	14.1	3.5	12.9–15.2	−0.21	0.760
eb-peb (mm)	15.2	5.0	13.6–16.9	14.5	5.4	12.7–16.3	−0.7	0.539
H-H′ (GnGo-H) (mm)	13.9	4.9	12.2–15.5	14.0	5.3	12.2–15.8	0.14	0.873

* *p*-value < 0.05 = Significance level.

**Table 3:** Cephalometric variables in control group at time T0 and T1, expressed as mean and standard deviation (SD) and their 95% confidence interval (95%CI).

Cephalometric Variables	Time						Mean Difference by Time	*p*-Value
	T0			T1			T1–T0	
	Mean	SD	95% CI	Mean	SD	95% CI		
SNA (°)	81.2	4.1	80.5–83.1	81.0	5.0	79.9–83.2	0.2	0.639
SNB (°)	78.4	4.3	77.0–79.7	78.3	4.7	77.6–79.8	0.1	0.809
ANB (°)	3.4	2.4	2.7–4.2	3.3	2.2	2.6–4.0	0.1	0.663
SN (mm)	61.3	5.1	59.6–62.9	63.2	7.0	60.9–65.4	−1.9	0.038
SN/MP (°)	34.5	5.5	32.7–36.3	35.4	7.2	33.1–37.7	−0.9	0.375
PP/MP (°)	28.4	5.4	26.6–30.1	27.7	4.8	26.1–29.3	0.6	0.260
P-A face height (S-Go/N-Me) (%)	63.8	4.9	62.2–65.4	64.0	4.3	62.6–65.4	−0.2	0.746
ad1-pns (mm)	15.6	6.5	13.5–17.7	16.7	5.0	15.1–18.3	−1.1	0.108
ad2-pns (mm)	8.6	4.0	7.3–9.9	9.2	3.2	8.1–10.2	−0.5	0.267
p-pp (mm)	11.3	4.6	9.9–12.8	10.7	4.6	9.2–12.1	0.7	0.281
pas-ppas (mm)	12.5	3.8	11.3–13.7	12.1	4.0	10.8–13.4	0.4	0.396
eb-peb (mm)	16.5	4.7	15.0–18.0	17.0	4.9	15.4–18.6	−0.4	0.624
H-H’ (GnGo-H) (mm)	11.0	5.1	9.4–12.7	10.3	5.5	8.5–12.1	0.7	0.299

No statistically significant differences were found in the control group.

## Data Availability

The data supporting the results of this study are available from the corresponding author upon reasonable request from any qualified investigator.
